# Origin and evolution of metal P-type ATPases in Plantae (Archaeplastida)

**DOI:** 10.3389/fpls.2013.00544

**Published:** 2014-01-07

**Authors:** Marc Hanikenne, Denis Baurain

**Affiliations:** ^1^Functional Genomics and Plant Molecular Imaging, Department of Life Sciences, Center for Protein Engineering (CIP), University of LiègeLiège, Belgium; ^2^PhytoSYSTEMS, University of LiègeLiège, Belgium; ^3^Eukaryotic Phylogenomics, Department of Life Sciences, University of LiègeLiège, Belgium

**Keywords:** P-type ATPases, paralogy, endosymbiosis, phylogenetics, evolution, metal transport, orthology

## Abstract

Metal ATPases are a subfamily of P-type ATPases involved in the transport of metal cations across biological membranes. They all share an architecture featuring eight transmembrane domains in pairs of two and are found in prokaryotes as well as in a variety of Eukaryotes. In *Arabidopsis thaliana*, eight metal P-type ATPases have been described, four being specific to copper transport and four displaying a broader metal specificity, including zinc, cadmium, and possibly copper and calcium. So far, few efforts have been devoted to elucidating the origin and evolution of these proteins in Eukaryotes. In this work, we use large-scale phylogenetics to show that metal P-type ATPases form a homogenous group among P-type ATPases and that their specialization into either monovalent (Cu) or divalent (Zn, Cd…) metal transport stems from a gene duplication that took place early in the evolution of Life. Then, we demonstrate that the four subgroups of plant metal ATPases all have a different evolutionary origin and a specific taxonomic distribution, only one tracing back to the cyanobacterial progenitor of the chloroplast. Finally, we examine the subsequent evolution of these proteins in green plants and conclude that the genes thoroughly characterized in model organisms are often the result of lineage-specific gene duplications, which calls for caution when attempting to infer function from sequence similarity alone in non-model organisms.

## Introduction

P-type ATPases constitute a superfamily of pumps using the energy of ATP to transport cations, and possibly phospholipids, across biological membranes (for detailed reviews, see Kuhlbrandt, 2004; Palmgren and Nissen, 2011). In phylogenetic analyses, P-type ATPases group according to protein architecture and substrate specificity, in both prokaryotes and Eukaryotes (Axelsen and Palmgren, 1998; Argüello, 2003; Hanikenne et al., 2005; Thever and Saier, 2009; Chan et al., 2010; Blaby-Haas and Merchant, 2012; Pedersen et al., 2012). Two (partially overlapping) classifications have been proposed for P-type ATPases. The first one is based on three architecture subtypes (AI–AIII). Proteins of architecture subtype AI have 8 transmembrane (TM) domains in pairs of two, whereas subtype AII proteins have 10 TM domains, and subtype AIII proteins display an unusual number of 7 TM domains (Thever and Saier, 2009; Chan et al., 2010). In the second classification system, the P-type ATPase superfamily is divided into five major classes, I–V, based on ion transport specificities and clustering in phylogenetic trees (Axelsen and Palmgren, 1998; Palmgren and Nissen, 2011).

PIB-type metal ATPases (or CPx metal ATPases) specifically transport metal cations. They share several structural features, including the presence of eight membrane-spanning domains (architecture subtype AI) and a conserved CPx motif in the sixth predicted TM domain, which is involved in metal cation translocation across the membrane (Axelsen and Palmgren, 1998; Argüello, 2003; Williams and Mills, 2005). PIB-type metal ATPases can be further divided into two groups: monovalent (Cu^+^/Ag^+^) and divalent (Zn^2+^/Cd^2+^/Pb^2+^/Hg^2+^/Cu^2+^) metal ATPases. Monovalent metal (Me^+^) ATPases correspond to subgroup IB-1, whereas divalent metal (Me^2+^) ATPases include the IB-2 and IB-4 subgroups, respectively, as described previously (Axelsen and Palmgren, 1998; Argüello, 2003).

Functionally, almost all metal ATPases pump metal out of the cytoplasm, i.e., out to the periplasm in prokaryotes and out of the cell or into an organelle (e.g., vacuoles, secretory vesicles, or chloroplasts) in Eukaryotes. This activity confers tolerance against toxic concentrations of metal and/or provides metals for metalloprotein maturation. Eight metal ATPases are found in the genome of the model plant *Arabidopsis thaliana* (Axelsen and Palmgren, 2001; Cobbett et al., 2003; Williams and Mills, 2005). Four of them are copper ATPases. (i) AtRAN1 is involved in copper delivery to the secretory pathway, where copper is required for the maturation of copper-dependent proteins (e.g., ethylene receptor, laccases) (Hirayama et al., 1999; Woeste and Kieber, 2000; Binder et al., 2010). (ii) AtHMA5 contributes to copper tolerance by controlling its accumulation in shoot (Andrés-Colás et al., 2006; Kobayashi et al., 2008). Polymorphisms in HMA5 coding sequences have been linked to natural variation in shoot copper accumulation among *A. thaliana* ecotypes (Kobayashi et al., 2008). AtRAN1 and AtHMA5 are related to ATP7A and ATP7B, the two human copper P-type ATPases that participate in the biosynthesis of copper-dependent proteins in the secretory pathway (e.g., caeruloplasmin). Mutations in these transporters are responsible for Menkes' and Wilson's diseases, respectively, both associated with cellular copper overload (Wang et al., 2011). (iii) AtPAA1 and AtPAA2 are chloroplastic proteins ensuring copper transport across the inner membrane and thylakoid membranes, respectively. Consistently, a *paa1* mutant is defective both in stromal Cu/ZnSOD and in plastocyanin, whereas the *paa2* mutant only lacks plastocyanin (Shikanai et al., 2003; Abdel-Ghany et al., 2005). The CtaA and PacS P-type ATPases found in the plasma membrane and thylakoid membrane of Cyanobacteria are respectively functional homologs of PAA1 and PAA2 proteins found in chloroplasts (Tottey et al., 2001).

The four additional metal ATPases found in *A. thaliana* transport divalent cations. Similarly to AtPAA1, AtHMA1 is localized in the inner membrane of the chloroplast and published data on both AtHMA1 and a barley homolog suggest that HMA1 is an exporter of copper (Cu^2+^), zinc, calcium, and cadmium out of the chloroplast (Seigneurin-Berny et al., 2006; Moreno et al., 2008; Kim et al., 2009; Mikkelsen et al., 2012). Finally, the AtHMA2–4 proteins are zinc and cadmium transporters. AtHMA2 and AtHMA4 are plasma membrane zinc and cadmium pumps and are involved in metal root-to-shoot translocation and redistribution (Hussain et al., 2004; Wong and Cobbett, 2009). AtHMA3 is localized in the vacuolar membrane and plays a role in zinc and cadmium tolerance (Morel et al., 2009). Polymorphism in AtHMA3 sequence explains most of the natural variation in cadmium leaf accumulation across *A. thaliana* accessions (Chao et al., 2012). Similarly, a functional homolog of AtHMA3 controls differential cadmium accumulation in grains of rice cultivars (Ueno et al., 2010; Miyadate et al., 2011).

Orthologs of both AtHMA3 and AtHMA4 are constitutively more highly expressed in *Arabidopsis halleri* and *Noccaea caerulescens*, two zinc and cadmium hyperaccumulators related to *A. thaliana* in the Brassicaceae (for recent reviews, see Verbruggen et al., 2009; Krämer, 2010; Hanikenne and Nouet, 2011) (see other contributions in this issue). Quantitative genetic analyses (Courbot et al., 2007; Willems et al., 2007; Frérot et al., 2010; Willems et al., 2010) and functional characterization established that high expression of *HMA4* is required for both hyperaccumulation and hypertolerance in *A. halleri* (Talke et al., 2006; Courbot et al., 2007; Hanikenne et al., 2008). Increased expression of *HMA4* in *A. halleri* occurred through a combination of copy number expansion (tandem triplication) and *cis*-regulatory changes activating the promoters of all three *HMA4* copies (Hanikenne et al., 2008). The *A. halleri HMA4* locus was shaped by positive selection, resulting in a selective sweep, and ectopic gene conversion, together substantiating selection for increased gene dosage (Hanikenne et al., 2013). In a fine example of parallel evolution, high expression of *HMA4* in *Noccaea caerulescens* was also found to result from copy number expansion and regulatory changes (O'Lochlainn et al., 2011; Craciun et al., 2012). In addition, differences in expression levels of both *HMA3* and *HMA4* were linked to variations in gene copy number between *N. caerulescens* populations exhibiting contrasted metal accumulation and tolerance (Ueno et al., 2011; Craciun et al., 2012).

If monovalent metal-transporting P-type ATPases are found in most organisms from the three domains of Life (Archaea, Bacteria, and Eukaryotes), the taxonomic distribution of divalent cation-transporting P-type ATPases is apparently limited to prokaryotes, a few algae and (land) plants (Axelsen and Palmgren, 1998; Argüello, 2003; Hanikenne et al., 2005; Thever and Saier, 2009; Chan et al., 2010; Blaby-Haas and Merchant, 2012; Pedersen et al., 2012). As metal ATPases play several key roles in metal homeostasis in plants, including the hyperaccumulation syndrome (see above), we conducted a robust phylogenetic analysis to determine their actual distribution across prokaryotes and Eukaryotes. We were keen to examine the origin of divalent metal P-type ATPases in Eukaryotes, especially how and when they appeared in the green lineage. Finally, we aimed to clarify orthology relationships among plant metal P-type ATPases, which is highly relevant when considering functional homology of proteins within and outside Brassicaceae.

## Methods

### Datasets

A taxonomically representative set of prokaryotic genomes was selected using the phylogenomic clustering method recently proposed by Moreno-Hagelsieb et al. (2013) (http://microbiome.wlu.ca/research/redundancy/). Using the GSSb model and a GSS threshold of 0.5, we retrieved 352 redundant clusters sorted according to the least overannotation criterion. A non-redundant prokaryotic dataset of 352 genomes corresponding to the best-annotated genome in each cluster was then assembled. With these clustering settings, cyanobacterial genomes grouped into three clusters and Cyanobacteria were thus represented by only three genomes in our non-redundant dataset. As we were particularly interested in analysing the ancestry of Plantae metal P-type ATPases, the 41 additional cyanobacterial genomes belonging to the three clusters were added to our non-redundant dataset. The complete genomes of a total of 393 prokaryotes were thus included in our study and downloaded from the NCBI FTP server in February 2013 (ftp://ftp.ncbi.nlm.nih.gov/genomes/Bacteria/) in the form of protein sequences (Table S1) (Benson et al., 2013).

For Eukaryotes, a total of 219 complete proteomes were downloaded from three different sources in February 2013: JGI (24 proteomes, http://genome.jgi.doe.gov/) (Grigoriev et al., 2012), Phytozome (41 proteomes, ftp://ftp.jgi-psf.org/pub/compgen/phytozome/v9.0/) (Goodstein et al., 2012), and Ensembl/Ensembl Genomes (154 proteomes, ftp://ftp.ensembl.org/pub/release-70/ and ftp://ftp.ensemblgenomes.org/pub/release-17/) (Kersey et al., 2012; Flicek et al., 2013) (Table S2).

EST sequences from 42 additional Plantae (Viridiplantae, Rhodophyta, and Glaucophyta) were downloaded in June 2013 from the NCBI EST database (http://www.ncbi.nlm.nih.gov/nucest/). Four additional, recently published, complete CDS datasets were also downloaded at that time from the respective lab websites (Table S3).

To annotate phylogenetic trees and to summarize the taxonomic distribution of the genes of interest, the NCBI Taxonomy database (Federhen, 2012) was weekly downloaded from the NCBI FTP server.

### HMM searches

HMM profiles were computed with hmmbuild and then used to query our complete proteomes with hmmsearch. hmmbuild and hmmsearch are components of the HMMER software package (version 3.0) (http://hmmer.org/) (see also Durbin et al., 1998). Specifically, the 826-match-state HMM profile representing all three architectures of prokaryotic P-type ATPases was computed from a 1656-AA alignment of the 82 Firmicutes proteins (e.g., *Bacillus*, *Staphylococcus*) described in Table 5 of Chan et al. (2010). The 1265-match-state HMM profile used to detect eukaryotic metal ATPases was computed from a 2978-AA alignment of all 873 metal ATPases identified in the non-redundant set of 393 prokaryotic proteomes.

### Alignments and phylogenetic analyses

#### Large trees (figures 1, 4, files s1–s3)

**Figure 1:**
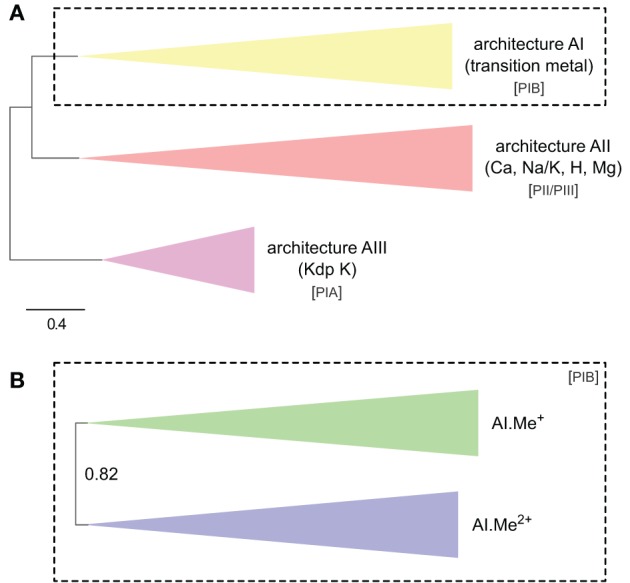
**Phylogeny of P-type ATPases (A) and metal ATPases in prokaryotes (B).** The two trees were obtained with PhyloBayes (C20 model) from the analysis of protein alignments of 1495 sequences × 295 unambiguously aligned amino acid (AA) positions **(A)** and 873 sequences × 361 AA **(B)**, respectively. In the schemes, tree branches were collapsed according to protein architecture **(A)** or metal specificity **(B)**. In **(A)**, the tree was rooted using the Kdp K family as outgroup, whereas the tree is unrooted in **(B)**. Statistical support is provided as posterior probabilities (PP). The scale bar at the bottom gives the number of substitutions per site. AI-AIII, architecture subtypes; Px, P-type ATPase subgroups (see text for details). The corresponding fully annotated trees are provided as NEXUS Files S1, S2, respectively.

Protein sequences were aligned through 10 iterations (–iter option) of Clustal Omega 1.1.0 using default settings (Sievers et al., 2011). The resulting alignments were filtered to eliminate poorly aligned positions and partial sequences using the Bio-MUST-Core software package (Denis Baurain, unpublished). Briefly, positions due to insertions in less than 50% of the sequences were discarded. Gblocks 0.91b (Castresana, 2000) was then used with either loose (Figure 1A and File S1) or medium (Figures 1B, 4 and Files S2, S3) parameters to further filter the least reliably aligned positions. Finally, for Figure 4 and File S3, the sequences having more than 50% missing characters with respect to the longest sequence were discarded.

The filtered alignments were analyzed using PhyloBayes 3.3e (Lartillot et al., 2009) under the C20 empirical profile mixture model, which leads to more accurate trees than ordinary empirical matrices (e.g., WAG or JTT) by accounting for site-specific amino-acid replacement patterns (Le et al., 2008). Each time, two independent chains were run for 1800–5500 cycles (depending on the dataset) until convergence of the various chain parameters (about 30–40 CPU days by chain). Statistical support is a built-in feature of Bayesian inference and is provided in the form of posterior probabilities (PP). Inferred tree topologies were largely congruent with those obtained in a maximum likelihood (ML) framework using RAxML 7.2.8 (Stamatakis, 2006) under the LG + F + Γ_4_ model (data not shown, Yang, 1993; Le and Gascuel, 2008). The LG model is an empirical matrix similar to WAG and JTT, but was estimated by incorporating the variability of evolutionary rates across sites.

#### Small trees (figures 7, 8, files s4–s7)

The protein sequences corresponding to the four eukaryotic metal ATPase subtrees were pruned from the alignment used in Figure 4. After automatic iterative re-alignment with Clustal Omega, each alignment was hand-curated using the editor of the MUST software package (Philippe, 1993). EST sequences were then added using the “forty” software package (Denis Baurain, unpublished) that completely automates EST database mining, contig assembly, and introduction of translated sequence fragments into existing protein alignments, while controlling for orthology relationships based on databases of paralogous sequences. The augmented alignments were once again hand-curated in the MUST editor (i) to selectively merge the multiple protein fragments belonging to a single gene from a single organism, (ii) to shorten (or even discard) the protein fragments with too many anomalous amino acids resulting from the translation of low-quality EST sequences, (iii) to locally improve the automated alignments carried out by forty, and (iv) to iteratively remove protein fragments that were too partial to be accurately positioned in the tree [as estimated from preliminary parsimony trees built using PAUP^*^ 4.0b10 (Swofford, 2002)]. Finally, the resulting alignments were filtered as above using Bio-MUST-Core to eliminate poorly aligned positions (Gblocks medium), except that no further filter for partial sequences was applied to avoid discarding informative protein fragments predicted from EST contigs.

ML trees were computed with PhyML 3.0 (Guindon et al., 2010) using the LG + F + Γ_4_ model. The starting tree for the heuristic search was computed by parsimony and the search included both NNI (nearest-neighbor interchange) and SPR (subtree pruning and regrafting) topological moves. Statistical support was estimated through the analysis of 100 bootstrap pseudo-replicates (Felsenstein, 1985).

### Sequence and tree annotations

HMMER reports and phylogenetic trees were automatically annotated for substrate specificity based on the best BLAST hit (Altschul et al., 1997) of each sequence against two small databases of annotated reference P-type ATPase sequences assembled from prokaryotes (Chan et al., 2010) or *A. thaliana* (Axelsen and Palmgren, 2001), respectively.

Tree rooting was done manually in Seaview (Gouy et al., 2010). Tree ladderization and annotation (e.g., substrate specificity, taxonomy, SPC/CPC motif), as well as clade coloring and collapsing at predetermined taxonomic levels, were automatically conducted using Bio-MUST-Core and further arranged in FigTree (http://tree.bio.ed.ac.uk/software/figtree/) running under Bio-Linux 7 (http://nebc.nerc.ac.uk/tools/bio-linux/) (Field et al., 2006). All annotated trees are provided as Files S1–S7 in NEXUS format, while the corresponding sequence alignments (before the Gblocks step) are given as a ZIP archive in Data Sheet S1.

### Sequence logos

Aligned sequences from Files S4–S7 were collected and the putative metal-binding regions regions corresponding to TM6 and TM7/8 were extracted. Then each of the eight sub-alignments was split into two parts, one with the green plants sequences (Viridiplantae) and the other with the remaining sequences of the corresponding tree (outgroup). Depending on the origin of the green plant genes, outgroup corresponded to other Plantae or Stramenopiles and/or to Cyanobacteria or Chlamydiae. Sequence logos were then computed using WebLogo 3.3 (Schneider and Stephens, 1990; Crooks et al., 2004), without compositional adjustment.

### Other analyses

All other figures (e.g., bubble and bar charts, 2-set Venn diagrams) were produced in R (R-Development-Core-Team, 2008) and further annotated using Inkscape (http://inkscape.org/). eulerAPE was used to generate the 3-set proportional Venn diagram (http://www.eulerdiagrams.org/eulerAPE/).

## Results

### Metal ATPases are a monophyletic group in prokaryotes

The full set of completely sequenced prokaryotic genomes (~2300 Bacteria and Archaea genomes on the NCBI server in February 2013) is too large to be readily amenable to phylogenetic analysis. Therefore, a clustering based on phylogenomic distance measures (Moreno-Hagelsieb et al., 2013) was used to assemble a non-redundant set of 352 complete genomes encompassing the whole taxonomic diversity of prokaryotes (see Methods). The set included 326 Bacteria (including 3 Cyanobacteria) and 26 Archaea (Table S1). As we were particularly interested in examining the ancestry of plant, algal, and cyanobacterial metal ATPases, 41 additional cyanobacterial genomes were added to this non-redundant genome set (Table S1).

To ascertain the relationships of metal ATPases within the P-type ATPase family among prokaryotes, we searched the proteins of our genome set with a hidden Markov model (HMM) profile computed from 82 Firmicutes P-type ATPases representing all three architecture subtypes found in prokaryotes (see Introduction, Chan et al., 2010). Proteins of architecture subtype AI correspond to metal ATPases (PIB). Proteins of architecture subtype AII correspond to calcium (PIIA), sodium/potassium (PIIC), proton (PIIIA), and magnesium (PIIIB) ATPases. Note that phospholipid flippases (PIV) are also part of subtype AII, but are only found in Eukaryotes (Axelsen and Palmgren, 1998; Chan et al., 2010). Finally, Kdp-potassium ATPases (PIA) belong to architecture subtype AIII (Chan et al., 2010). We elected to use HMM profiles rather than specific BLAST searches both to maximize detection sensitivity and to minimize sampling biases that might be caused by non-random selection of a limited number of query sequences.

**Figure 2:**
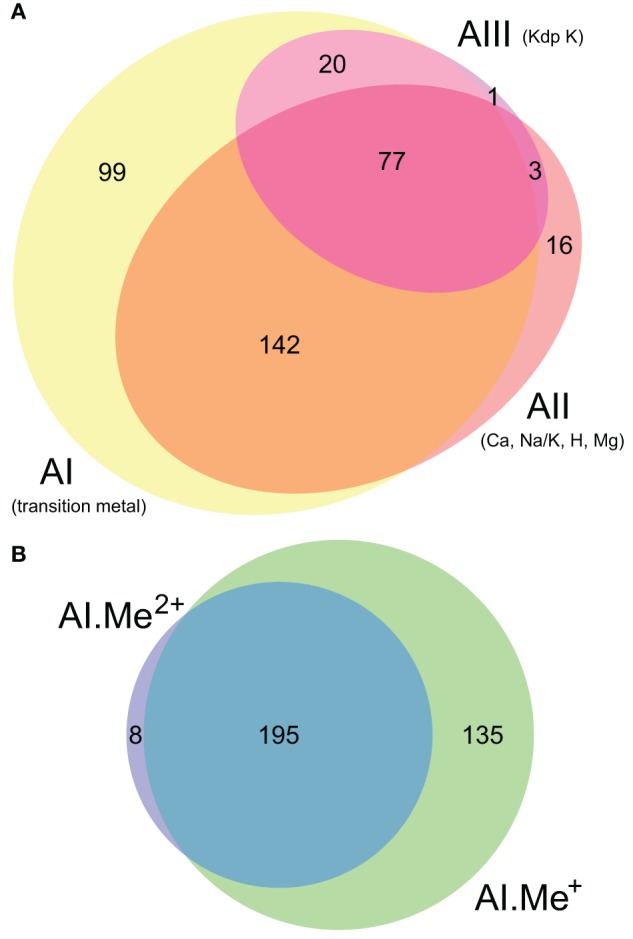
**Co-occurrence statistics for the three architectures (AI–AIII) of P-type ATPases (A) and for Me^+^ (Cu, Ag) and Me^2+^ (Zn, Cd…) ATPases (B) in prokaryotic genomes, respectively.** Areas are proportional to the number of individual genomes containing a given protein subtype. In **(B)**, architecture AI proteins were further divided into two groups (AI.Me^+^ and AI.Me^2+^) based on metal specificity.

**Figure 3:**
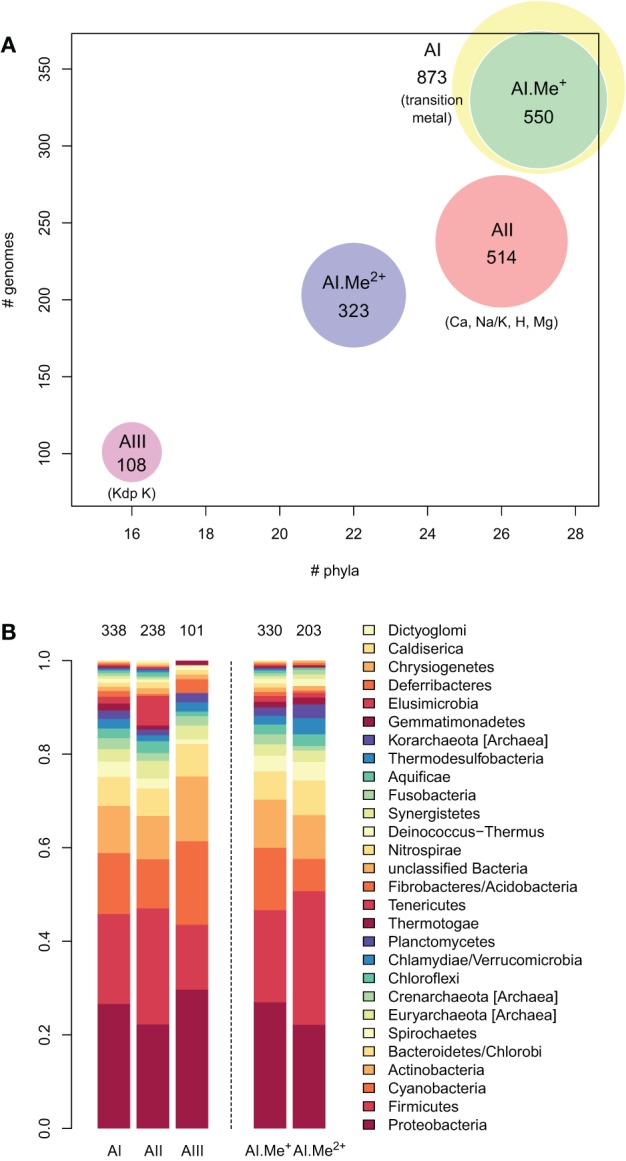
**Taxonomic distribution of the three architectures (AI–AIII) of P-type ATPases and Me^+^ (Cu, Ag) and Me^2+^ (Zn, Cd…) ATPases in prokaryotes. (A)** Each bubble is located in the graph according to the number of genomes (*y*-axis) and phyla (*x*-axis) containing each architecture subtype. The bubble areas are proportional to the total number of proteins of a given architecture type identified in the prokaryotic dataset. The AI bubble in yellow corresponds to the sum of the AI.Me^+^ (green) and AI.Me^2+^ (blue) bubbles, since these are two subgroups of the former. **(B)** Relative distribution of each architecture type among prokaryotic phyla (according to NCBI Taxonomy). The total numbers of individual genomes containing each architecture subtype are given on top of the bars. Architecture AI proteins were further divided into two groups (AI.Me^+^ and AI.Me^2+^) based on metal specificity.

In the HMM search report, we observed a breakdown in *E*-values around 1e-20, which allowed distinguishing P-type ATPases from other ATP-binding protein families with high specificity. At this *E*-value threshold, we retrieved a total of 1495 proteins from 336 Bacteria and 23 Archaea, respectively (Table S1). The 1495 proteins were then aligned and subjected to Bayesian phylogenetic inference, using an evolutionary model that allows for different functional constraints across protein sites (see Methods). For easy interpretation, the resulting phylogenetic tree (Figure 1A, File S1) was annotated with both substrate specificity and taxonomic information (see Methods). Prokaryotic P-type ATPases robustly clustered into three groups, corresponding to the three protein architectures (Figure 1A). Among these, metal ATPases are monophyletic (i.e., formed a discrete and homogeneous group) and represent the most common P-type ATPases found in Bacteria and Archaea (Figures 1A, 2A, 3), in agreement with previous findings (Argüello, 2003; Chan et al., 2010; Palmgren and Nissen, 2011).

To dissect relationships between Me^+^ and Me^2+^ ATPases in our prokaryotic dataset, the 873 metal ATPases were extracted from the tree, realigned and once more subjected to Bayesian phylogenetic inference (Figure 1B, File S2). Notably, the tree featured a fairly supported branch (posterior probability or *PP* = 0.82) separating Me^+^ and Me^2+^ ATPases, which suggests that cation selectivity is a conserved property among these proteins. Although, we cannot rule out that one of these two groups might be paraphyletic (i.e., ancestral to the other, more derived, one), we favor the hypothesis of two monophyletic groups based on their wide taxonomic distribution (Figure 3B), in agreement with Chan et al. (2010). This means that Me^+^ and Me^2+^ ATPases much likely stem from the duplication of an ancestral metal ATPase early in the evolution of Life. Note however that Me^+^ ATPases are more common than Me^2+^ ATPases in extant prokaryotes (Figures 2B, 3A).

### Origin of eukaryotic metal ATPases

As prokaryotic metal ATPases are monophyletic, a single HMM profile was computed from the 873 metal ATPases described above and used to search the complete proteomes of 219 Eukaryotes, which covered a good share of eukaryotic diversity (including e.g., Opisthokonts, Plantae, and Stramenopiles; for a full list see Table S2).

**Figure 4:**
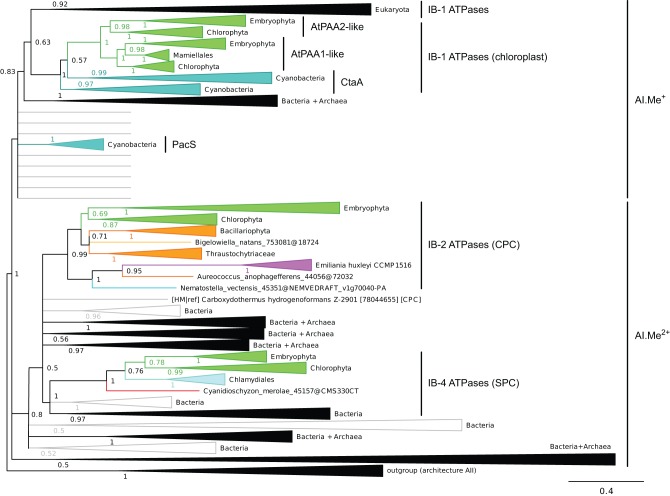
**Phylogeny of IB metal ATPases in prokaryotes and Eukaryotes.** The tree was obtained with PhyloBayes (C20 model) from the analysis of a protein alignment of 1761 sequences × 340 AA. Tree branches were colored based on homogeneous taxonomic composition and collapsed to highlight relationships between prokaryotic and eukaryotic proteins. The tree was rooted using a small group of architecture AII proteins as outgroup. Statistical support is provided as PP. The scale bar at the bottom gives the number of substitutions per site. The corresponding fully annotated tree is provided as NEXUS File S3.

Each protein in the HMM search report was further annotated for substrate specificity based on similarity with *A. thaliana* P-type ATPases (described in Axelsen and Palmgren, 2001). At an *E*-value threshold of 1e-78, we retrieved a total of 942 proteins from 216 species corresponding mostly to proteins annotated as Me^+^ and Me^2+^ ATPases (Table S2). Metal ATPases were thus found in all eukaryotic proteomes but three (one Archamoebae, *Entamoeba histolytica*, and two Metazoa, *Choloepus hoffmanni*, *Nasonia vitripennis*, respectively), but these exceptions might be due to extreme sequence divergence or genome mis-annotation. The 942 eukaryotic proteins were aligned with the 873 prokaryotic metal ATPases described above. To minimize artifacts during phylogenetic reconstruction, 54 conspicuously short protein sequences (50 from Eukaryotes and 4 from Bacteria), mostly corresponding to incomplete splice variants or mis-modeled proteins, were discarded from the alignment (see Methods). A large phylogenetic tree was then obtained by Bayesian inference (Figure 4, File S3). After addition of eukaryotic metal ATPases, Me^+^ and Me^2+^ ATPases again clustered into two distinct groups, thus further supporting the conclusions drawn from Figure 1B. Altogether, 665 Me^+^ ATPases and 218 Me^2+^ ATPases were identified in Eukaryotes (Table S2). A group of nine proteins corresponding to calcium ATPases were also present in the set (designed as outgroup in Figure 4). They were not included in further analyses.

**Figure 5:**
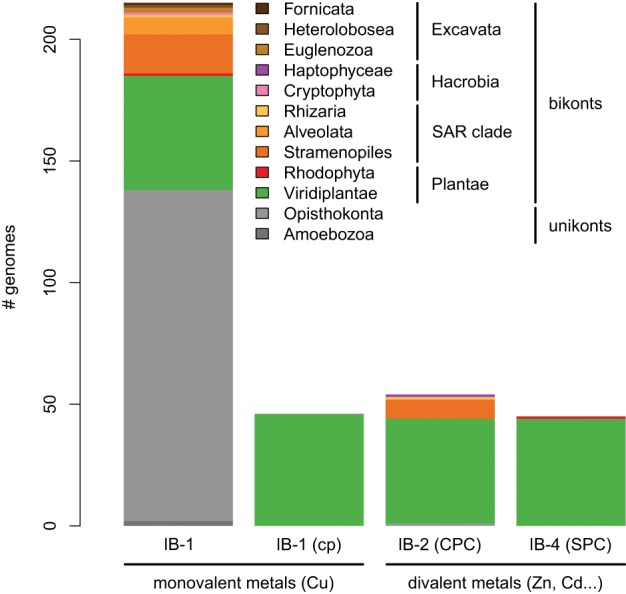
**Taxonomic distribution of the four subgroups of metal ATPases in Eukaryotes.** The *y*-axis gives the number of genomes containing at least one protein of a given subgroup.

We next examined the relationships among eukaryotic and prokaryotic metal ATPases in the tree and observed that four subgroups of eukaryotic proteins had distinct evolutionary origins (Figure 4). First, eukaryotic metal ATPases clustering with prokaryotic Me^+^ ATPases resolved into two subgroups, both being part of IB-1 P-type ATPases defined by Axelsen and Palmgren (1998):

- Eukaryotic Me^+^ ATPases involved in copper pumping into the secretory pathway or in copper exclusion from cells all clustered together. Hence, ATP7A and ATP7B fell within a large clade of Opisthokont proteins, as expected (Wang et al., 2011). Similarly, Plantae proteins involved in related cellular functions (e.g., HMA5 and RAN1 in *A. thaliana*) fell within this subgroup as well (Table 1 and File S4). In fact, these Me^+^ ATPases were recovered in all but four eukaryotic genomes included in our dataset (Figure 5, Table S2) and accounted for the vast majority of eukaryotic Me^+^ ATPases. Since this subgroup is moreover not associated to any particular prokaryotic phyla, it corresponds to the eukaryotic counterpart of prokaryotic Me^+^ ATPases, which likely retained the ancestral function tracing back to the common ancestor of prokaryotes and Eukaryotes.
- Eukaryotic chloroplast Me^+^ ATPases involved in copper transport into chloroplasts were found in Viridiplantae only (i.e., land plants and green algae) (Figure 4, Table 1) and clustered separately from the first subgroup described above. Even though our Bayesian tree suggests that these two eukaryotic subgroups might be quite close in the prokaryotic diversity, this result is not compelling because the statistical support is weak (*PP* = 0.63) and because this association was not recovered when analysing the same alignment in a ML framework under another model (not shown). Chloroplast Me^+^ ATPases provide copper for incorporation in the electron carrier plastocyanin and/or copper/zinc superoxide dismutases (Nouet et al., 2011). The presence of two copper ATPases in chloroplasts, one in the inner membrane (e.g., PAA1 in *A. thaliana*) (Shikanai et al., 2003) and one in the thylakoid membrane (e.g., PAA2 in *A. thaliana*) (Abdel-Ghany et al., 2005), is an ancestral feature of Viridiplantae (see also Merchant et al., 2006; Blaby-Haas and Merchant, 2012). These two paralogous proteins are orthologous to the lone cyanobacterial CtaA copper ATPase (Figure 4), thus indicating acquisition through primary endosymbiosis, followed by a duplication of the original gene. In Cyanobacteria, CtaA is localized in the plasma membrane, carrying out copper import into the cell, and is required for plastocyanin assembly (Phung et al., 1994; Tottey et al., 2001). In contrast, copper transport into cyanobacterial thylakoids is ensured by the PacS copper ATPase (Kanamaru et al., 1994; Tottey et al., 2001). Our tree shows that PacS proteins are not related to eukaryotic chloroplast Me^+^ ATPases, but are part of the diversity of prokaryotic copper ATPases (Figure 4), in agreement with Williams and Mills (2005). The chloroplast copper uptake system thus represents a nice example of gene duplication followed by neofunctionalization to maintain function: the CtaA and PacS proteins found in the plasma membrane and thylakoid membrane of Cyanobacteria are functional homologs of PAA1-like and PAA2-like proteins found in chloroplasts, respectively, but both eukaryotic proteins are co-orthologs of CtaA only (Figure 4). Finally, note that chloroplast Me^+^ ATPases are missing in *Cyanidioschyzon merolae* (an early-branching red alga), which is also lacking plastocyanin, as previously described (Hanikenne et al., 2005).

**Table 1:**
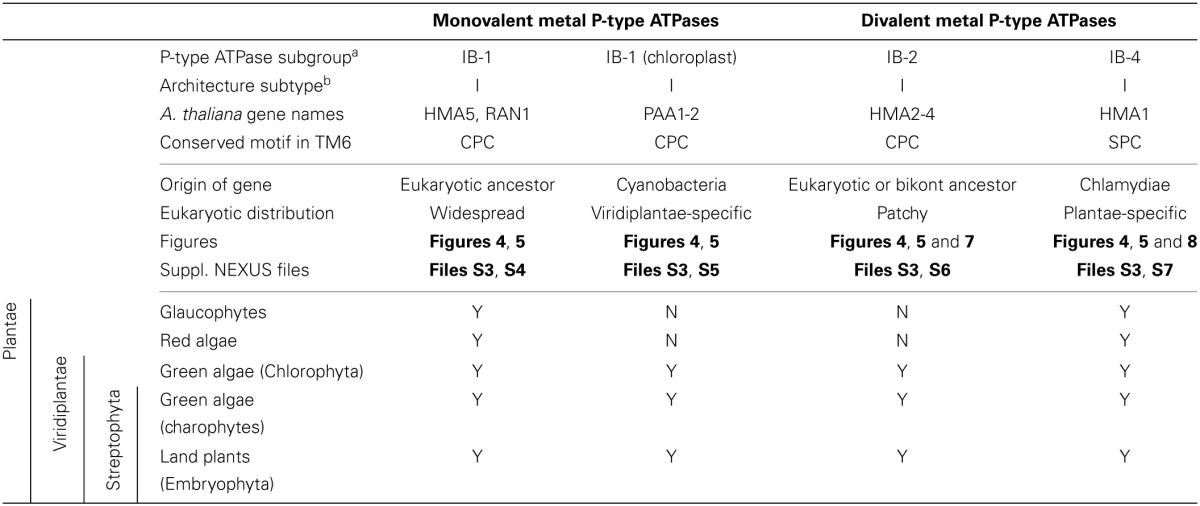
**Overview of origin and taxonomic distribution of IB metal P-type ATPases in Eukaryotes**.

^a^According to Axelsen and Palmgren (1998), Argüello (2003).

^b^According to Thever and Saier (2009), Chan et al. (2010).

Our phylogenetic analysis thus suggests that IB-1 P-type ATPases defined by Axelsen and Palmgren (1998) could be further divided into two subgroups, as described above, to take into account our observation that the additional eukaryotic IB-1 ATPases found in chloroplasts have a distinct origin with respect to the bulk of eukaryotic IB-1 ATPases.

**Figure 6:**
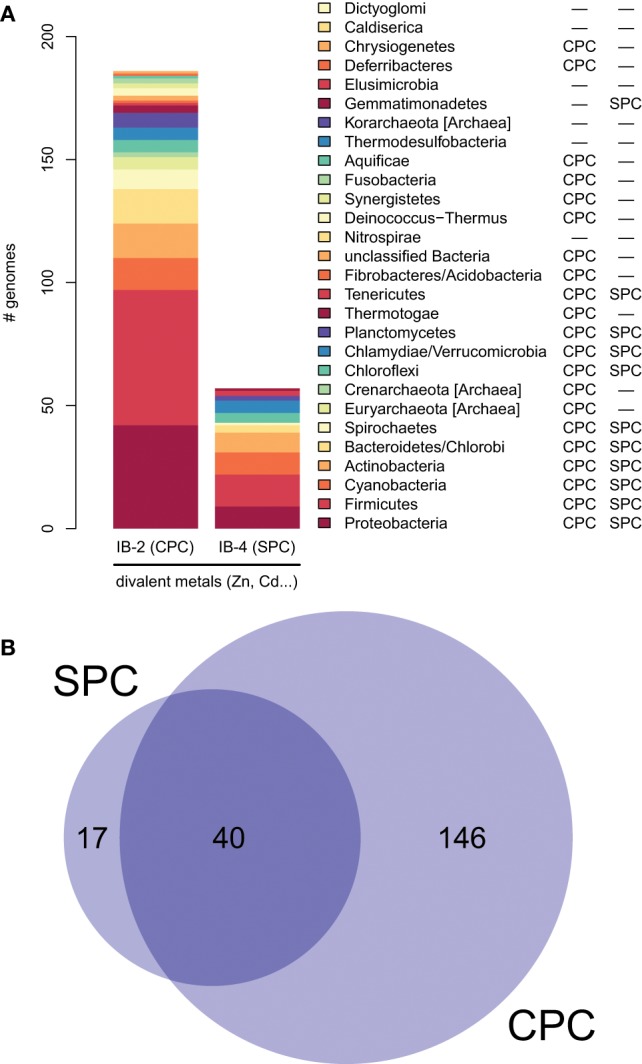
**Taxonomic distribution of IB-2 (CPC) and IB-4 (SPC) Me^2+^ ATPases in prokaryotes. (A)** The *y*-axis gives the number of genomes containing at least one protein of a given subgroup. The two columns on the right list a qualitative taxonomic breakdown of the co-occurrence statistics shown in **(B)**, in which areas are proportional to the number of individual genomes containing a given protein type.

Second, a more limited number of 218 eukaryotic P-type ATPases clustered with prokaryotic Me^2+^ ATPases (Figure 4, Table S2). These Me^2+^ ATPases displayed a narrower taxonomic distribution than Me^+^ ATPases and were found in only 58 species (Figure 5, Table S2). They resolved into two subgroups corresponding to IB-2 and IB-4 P-type ATPases described by Axelsen and Palmgren (1998). As for Me^+^ ATPases, we observed that each subgroup of Me^2+^ ATPases had a distinct evolutionary origin:

- Subgroup IB-2 of Me^2+^ ATPases, which corresponds to the HMA2, HMA3, and HMA4 proteins of *A. thaliana* (Figure 4), had a patchy taxonomic distribution among mostly photosynthetic organisms: land plants (embryophytes), green algae (chlorophytes), Stramenopiles [diatoms, pelagophytes (*Aureococcus anophagefferens*) and non-photosynthetic thraustochytrids], chlorarachniophytes (*Bigelowiella natans*) and coccolithophorid haptophytes (*Emiliania huxleyi*) (Figures 4, 5, Table 1). Surprisingly, one Me^2+^ ATPase was also found in the starlet sea anemone (*Nematostella vectensis*). Considering our broad sampling of animal proteomes, the latter is probably the result of a horizontal gene transfer (HGT) or of a contamination during genome sequencing.
  Proteins of this subgroup have the canonical CPx (mostly CPC) motif of metal ATPases in the sixth predicted transmembrane domain (Figures 4, 6; see also Figure 9).
  Based on a partial view of the taxonomic distribution of this subgroup (mostly in primary photosynthetic species, Bacteria and Archaea but excluding most Eukaryotes), it has been so far assumed that the IB-2 Me^2+^ pumps found in plants evolved from chloroplast proteins acquired from the cyanobacterial endosymbiont, and were later recruited to the plasma membrane or to the vacuole (Cobbett et al., 2003; Pedersen et al., 2012). Our phylogenetic analysis suggests otherwise, though. Since eukaryotic IB-2 Me^2+^ ATPases are no more related to extant Cyanobacteria than to the whole prokaryotic diversity of Me^2+^ ATPases (Figure 4), this means that these proteins were not introduced into photosynthetic Eukaryotes by the primary endosymbiosis. Instead, we propose the following hypothesis: IB-2 Me^2+^ ATPases fulfil an ancient function, tracing back to the common ancestor of prokaryotes and Eukaryotes, and their genes experienced multiple parallel losses among eukaryotic lineages. Such a scenario would explain their patchy distribution, which is not restricted to Plantae or even to photosynthetic organisms (thraustochytrids are non-photosynthetic Stramenopiles). Recent HGT between Eukaryotes cannot be fully ruled out but appears unlikely in this case because green plant sequences are monophyletic in our tree. Similarly, sodium/potassium (Na/K) P-type ATPases are present in prokaryotes and animals but not in plants (Palmgren and Nissen, 2011).
- Subgroup IB-4 of eukaryotic Me^2+^ ATPases includes proteins related to the *A. thaliana* HMA1 protein (Figure 4), which is localized in the chloroplast (Seigneurin-Berny et al., 2006; Kim et al., 2009; Mikkelsen et al., 2012). It is specific to Plantae (or Archaeplastida, i.e., green plants, red algae, and glaucophytes) (Figures 4, 5, Table 1). However, this subgroup clustered with proteins belonging to the bacterial phylum Chlamydiae, and not with Cyanobacteria, as usually observed for Plantae-specific proteins. HMA1-like proteins are in fact part of a small group of about 50 genes, essentially linked to chloroplastic functions that are thought to result from direct HGT from Chlamydiae into the common ancestor of Plantae (Huang and Gogarten, 2007; Moustafa et al., 2008; Baum, 2013). Thus, IB-4 Me^2+^ ATPases have an origin that is both very clear and completely distinct from that of the IB-2 subgroup of eukaryotic Me^2+^ ATPases (e.g., AtHMA2, −3, and −4).
  Moreover, proteins of the IB-4 subgroup share an uncharacteristic Ser/Pro/Cys (SPC) motif in the sixth predicted transmembrane domain instead of the common Cys-Pro-Cys/His/Ser (CPx) motif characteristic of all other metal P-type ATPases (see also Argüello, 2003; Pedersen et al., 2012). An exception is represented by a paraphyletic subset of chlorophytes (e.g., *Ostreococcus*, *Micromonas*, *Coccomyxa*) that possess an even more unusual APC motif, whereas Volvocales green algae (e.g., *Chlamydomonas*) and the red alga *C. merolae* display the SPC motif (Figure 4, see also Hanikenne et al., 2005). A switch from a CPx motif to a SPC or APC motif may alter metal specificity of the transporters within this subgroup (Cobbett et al., 2003; Hanikenne et al., 2005; Williams and Mills, 2005) and may be related to the broader metal specificity reported for AtHMA1 compared to AtHMA2–4 (see Introduction). Me^2+^ ATPases of 57 Bacteria (including Chlamydiae) are strongly associated (*PP* = 1) with this IB-4 subgroup of eukaryotic Me^2+^ ATPases. As expected, they also all present the SPC motif (Figures 4, 6; see also Figure 9), except three species that show the APC motif instead. The CPx motif, which is also present in Me^+^ ATPases, corresponds to the ancestral state (Figure 6). IB-4 Me^2+^ ATPases (SPC) are absent in Archaea and in (supposedly) early-branching Bacteria (Thermotogae, Aquificae, and Fusobacteria). Therefore, following the terminology proposed by Battistuzzi and Hedges (2009), IB-4 Me^2+^ ATPases (SPC) possibly derived from IB-2 Me^2+^ ATPases (CPx) after a gene duplication having affected the common ancestor of Hydrobacteria and Terrabacteria. Secondary loss of IB-2 or IB-4 Me^2+^ ATPases then independently occurred in several lineages (Figure 6).

### Evolution of metal ATPases in plantae

Plantae are a monophyletic group of Eukaryotes stemming from the primary endosymbiosis. It includes glaucophytes, red algae (rhodophytes), and green plants (Viridiplantae) (Cavalier-Smith, 1981; Rodriguez-Ezpeleta et al., 2005). Green plants are further split into two major lineages, chlorophytes and streptophytes. While chlorophytes contain only green algae, streptophytes include six lineages of green algae (commonly referred to as charophytes) and a seventh lineage corresponding to land plants (embryophytes) (Bremer, 1985; Lewis and McCourt, 2004; Laurin-Lemay et al., 2012). Depending on the availability of complete genomes, our initial eukaryotic set only represented a limited diversity of Plantae. Hence, it included no glaucophyte, a single red alga, only micro-chlorophytes (e.g., *Ostreococcus*, *Micromonas*, *Chlamydomonas*) and no charophyte. Similarly, completely sequenced genomes of land plants do not yet cover their whole diversity, and many important groups (e.g., gymnosperms) were missing from our initial analyses.

To better study the evolutionary trajectory of the four metal ATPase subgroups described in the previous section, we mined EST databases and newly available complete proteomes from 44 additional Plantae (Table S3). EST inclusion into the general metal ATPase alignment of Figure 4 followed a careful procedure aimed at ensuring that only true orthologs were retained for further analyses (see Methods). This resulted in the addition of 71 proteins from 24 Plantae, whereas the remaining 20 EST datasets did not yield any metal ATPase, generally because of their limited amount of sequences.

For each of the four metal ATPase subgroups, an enriched alignment was then built and thoroughly hand-curated before being subjected to ML inference (see Methods; Files S4–S7). Note that the four trees obtained were only used to examine presence/absence of the various subgroups at the phylum level. We did not attempt to assemble an inventory of those proteins in the different species because EST mining often yields partial and non-overlapping protein sequences, which do not represent the whole gene complement of the corresponding organism.

**Figure 7:**
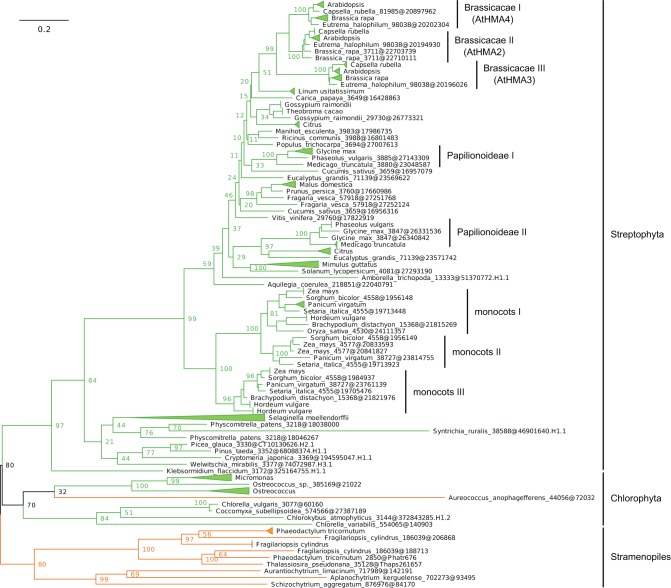
**Phylogeny of IB-2 Me^2+^ ATPases in Eukaryotes.** The tree was obtained with PhyML (LG + F + Γ_4_ model) from the analysis of a protein alignment of 138 sequences × 496 AA. The tree was rooted using Stramenopiles as outgroup and, for clarity, branches were collapsed at the genus level. Bootstrap proportions for selected nodes (mostly at family level or above) are shown. The scale bar at the bottom gives the number of substitutions per site. Note that *Aureococcus anophagefferens* (Stramenopiles, Pelagophyceae) is located among chlorophytes, possibly due to a long-branch artifact (Felsenstein, 1978). The corresponding NEXUS file is provided as File S6.

**Figure 8:**
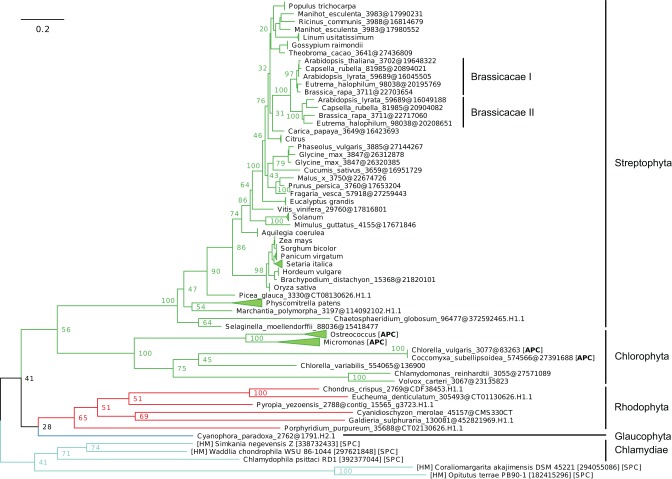
**Phylogeny of IB-4 Me^2+^ ATPases in Eukaryotes.** The tree was obtained with PhyML (LG + F + Γ_4_ model) from the analysis of an alignment of 91 sequences × 563 AA. The tree was rooted using the Chlamydiae/Verrumicrobia as outgroup and, for clarity, branches were collapsed at the genus taxonomic level. Bootstrap proportions for selected nodes (mostly at family level or above) are shown. The scale bar at the bottom gives the number of substitutions per site. The corresponding NEXUS file is provided as File S7.

Taxonomic distribution of the four subgroups within Plantae was as follows:

- Me^+^ ATPases were found in all Plantae phyla, thus confirming their broad taxonomic distribution in Eukaryotes (Table 1, File S4). The observation of the tree revealed a complex evolutive history in Plantae. From a single ancestral protein, as found in extant chlorophytes, two gene duplication events occurred during the evolution of streptophytes: the first before the colonization of land and the second later, probably in the spermatophyte (angiosperms and gymnosperms) ancestor (File S4). A gene copy was later lost in Brassicaceae, resulting in the presence of only two proteins (e.g., AtHMA5 and AtRAN1) in this family. Note that gene duplication events also occurred in chlorophytes after the split from streptophytes (e.g., in *C. reinhardtii*, see Blaby-Haas and Merchant, 2012).
- Chloroplast Me^+^ ATPases are present in Viridiplantae only, which suggests secondary loss in both glaucophytes and rhodophytes. Such a loss may be linked to the absence of the major copper-requiring protein plastocyanin since, in glaucophytes and rhodophytes, transfer of photosynthetic electrons from cytochrome *b*_6_/*f* to PSI is carried out by cytochrome *c*_6_ (Matsuzaki et al., 2004; Price et al., 2012). However, the presence of two chloroplast copper ATPases is a shared feature of all Viridiplantae (File S5).
- Within Plantae, IB-2 Me^2+^ ATPases are specific to Viridiplantae and were found in chlorophytes, charophytes, and land plants (Figure 7, File S6). In angiosperms, several independent gene duplication events occurred in different families. For example, a triplication specific to Brassicaceae resulted in the presence of three terminal paralogs (e.g., AtHMA2–4), whereas other gene duplications or triplications took place in Papilionoideae and monocots, respectively (Figure 7).
- Finally, IB-4 Me^2+^ ATPases (SPC) are present in all Plantae, where a single protein of this type was found in most species (Figure 8). These proteins are thus orthologous across Bacteria (including the Chlamydiae donor) and Plantae. Note that a gene duplication occurred in Brassicaceae, followed by a gene loss in *A. thaliana* only, resulting in the presence of a single HMA1 in this species.

### Functional conservation

**Figure 9:**
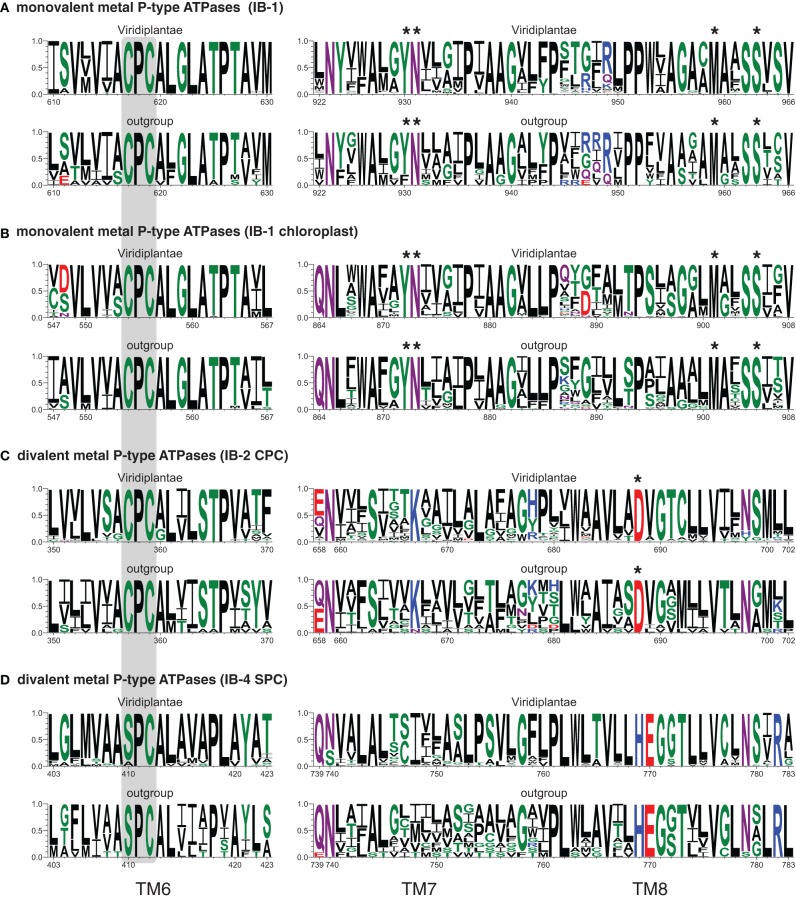
**Sequence logos of TM domains 6–8 of the four subgroups of metal P-type ATPases (A–D).** The height of each letter gives the occurrence frequency of the corresponding amino-acid residue across the aligned sequences (*y*-axes), without compositional adjustment for simplicity. *x*-axes are numbered according to the corresponding *A. thaliana* proteins: AtHMA5 **(A)**, AtPAA1 **(B)**, AtHMA4 **(C)**, AtHMA1 **(D)**. The CPx/SPx motif is on gray background, while an asterisk designates the amino-acid residues whose mutations inactivate the enzymes. The number of sequences used for computing each logo was as follows: 224 (A-Viridiplantae), 13 (A-outgroup: Glaucophyta and Rhodophyta), 133 (B-Viridiplantae), 43 (B-outgroup: Cyanobacteria), 124 (C-Viridiplantae), 13 (C-outgroup: Stramenopiles), 79 (D-Viridiplantae), 12 (D-outgroup: Chlamydiae, Glaucophyta and Rhodophyta).

Conserved amino acids, mostly with polar side chains, were identified in TM domains 6, 7, and 8 of PIB-type ATPases. Forming putative TM metal binding domains, these invariant amino acids may play a role in determining metal specificity of the pumps (Argüello, 2003; Argüello et al., 2007). To examine the conservation of these motifs in the four IB subgroups in extant green plant sequences (Viridiplantae) (Figures 7, 8, Files S4–S7), we computed sequence logos for the TM6 and TM7–8 regions (Figure 9). The remaining sequences in each corresponding tree were used as outgroups, which are the closest available proxies to the ancestral sequences that were at the base of each PIB-type ATPase subgroup. IB-1 and IB-1 chloroplast copper ATPases all share a CPC motif in TM6, and the presence of invariant YN (TM7) and M, SS (TM8) amino acids (Figures 9A,B). These are characteristic of copper transporters, although Y → F substitutions were observed in the outgroup (Figure 9A). Mutations in those motifs inactivate the enzyme (Mandal et al., 2004) or have been linked to Wilson's disease (Argüello et al., 2007).

K (TM7) and D (TM8) residues are conserved in IB-2 Me^2+^ ATPases (Figure 9C). Mutations in the D residue of ZntA of *E. coli* inactivate the pump, suggesting that this residue could be involved in Zn coordination (Dutta et al., 2006). Finally, a HEGxT motif in TM8 is shared by IB-4 Me^2+^ ATPases, in addition to the APC/SPC motif in TM6, although the function of these residues has not been investigated yet (Figure 9D).

In our sequence logos, additional conserved positions (shared between Viridiplantae and their outgroups) can be specifically observed for each of the four subgroups; these residues may also play a role in metal specificity (Figure 9).

## Discussion

Our phylogenetic analyses based on a taxonomically representative set of prokaryotic genomes and on all annotated eukaryotic genomes confirmed that metal ATPases are monophyletic and can be further divided into two groups, Me^+^ and Me^2+^, based on substrate specificities (Figure 1) (see Axelsen and Palmgren, 1998; Argüello, 2003; Chan et al., 2010). In Eukaryotes, metal ATPases clustered into four subgroups scattered in the prokaryotic diversity. Observation of the relationships among the four subgroups of eukaryotic metal ATPases and with their prokaryotic counterparts suggested that they had diverse evolutionary origins, an interpretation that was strengthened by the analysis of the taxonomic composition of these four subgroups (Figure 5, Table 1).

IB-1 Me^+^ ATPases are very common (Figures 3, 5) and possibly represent an ancestral function tracing back to LUCA (Last Universal Common Ancestor, the most recent ancestor of extant living organisms) (e.g., Koonin, 2010). Common to all Viridiplantae, IB-2 Me^2+^ ATPases displayed a patchy distribution in other Eukaryotes, appearing to be limited to some bikont lineages (Figure 5) (Stechmann and Cavalier-Smith, 2002). Eukaryotic IB-2 Me^2+^ ATPases are clearly monophyletic, which rules out multiple recruitments from prokaryotes during eukaryotic evolution (from Cyanobacteria in Viridiplantae for example), and are equally related to the whole diversity of their prokaryotic homologues, thus suggesting that they also trace back to LUCA (or at least to the bikont ancestor). Their patchy distribution in Eukaryotes would then result from multiple independent losses.

Chloroplast IB-1 Me^+^ ATPases and IB-4 Me^2+^ ATPases are restricted to Viridiplantae and Plantae, respectively (Figure 5, Table 1). Chloroplast IB-1 Me^+^ ATPases were acquired from a cyanobacterial ancestor in the course of primary endosymbiosis (Figure 4, File S5), whereas IB-4 Me^2+^ ATPases arose from a HGT event from Chlamydiae into the Plantae ancestor (Archaeplastida) (Figures 4, 8).

Looking at detailed phylogenetic analyses allow disentangling the complex orthology and co-orthology relationships between Plantae metal ATPases, which cannot be achieved through mere similarity searches (e.g., BLAST). In addition to the example of the two Viridiplantae paralogs of chloroplast copper ATPases originating from a single cyanobacterial protein ancestor (see above), our analyses revealed that several gene duplication events occurred within IB-1 Me^+^ and IB-2 Me^2+^ ATPases in Plantae. These gene duplications, some of them associated to whole genome duplication events (e.g., Van De Peer et al., 2009), were often restricted to specific lineages (e.g., a triplication resulting in the presence of HMA2, HMA3 and HMA4 paralogs in Brassicaceae). This highlights that the generalization of functional data/hypotheses obtained in *A. thaliana* to plants outside Brassicaceae has to be done with care.

Altogether, our phylogenetic analyses shed light on the evolution of metal ATPases in Eukaryotes, with a particular focus on Plantae. It also provides a solid phylogenetic framework for their functional analyses outside model plant species.

## Conflict of interest statement

The authors declare that the research was conducted in the absence of any commercial or financial relationships that could be construed as a potential conflict of interest.

## Supplementary material

Table S1 **Overview of prokaryotic genomes included in the study.** The number of P-type ATPases identified in each genome is provided for each architecture subtype (AI–AIII) and, for architecture AI, for each of monovalent metal (AI.Me^+^) and divalent metal (AI.Me^2+^) groups.

Table S2 **Overview of eukaryotic genomes included in the study.** The number of monovalent metal (AI.Me^+^) and divalent metal (AI.Me^2+^) ATPases identified in each genome is provided.

Table S3 **Overview of additional ESTs and genomes included in the study**.

File S1 **Phylogenetic tree of P-type ATPases in prokaryotes (NEXUS file)**.

File S2 **Phylogenetic tree of IB metal ATPases in prokaryotes (NEXUS file)**.

File S3 **Phylogenetic tree of IB metal ATPases in prokaryotes and Eukaryotes (NEXUS file)**.

File S4 **Phylogenetic tree of IB-1 Me^+^ ATPases in Plantae (NEXUS file)**.

File S5 **Phylogenetic tree of IB-1 chloroplast Me^+^ ATPases in Cyanobacteria and Viridiplantae (NEXUS file)**.

File S6 **Phylogenetic tree of IB-2 Me^2^+ ATPases in Stramenopiles and Viridiplantae (NEXUS file)**.

File S7 **Phylogenetic tree of IB-4 Me^2^+ ATPases in Chlamydiae and Plantae (NEXUS file)**.

DataSheet1 **Alignments in FASTA format for Files S1–S7**.
